# Severe Anemia in Papua New Guinean Children from a Malaria-Endemic Area: A Case-Control Etiologic Study

**DOI:** 10.1371/journal.pntd.0001972

**Published:** 2012-12-13

**Authors:** Laurens Manning, Moses Laman, Anna Rosanas-Urgell, Pascal Michon, Susan Aipit, Cathy Bona, Peter Siba, Ivo Mueller, Timothy M. E. Davis

**Affiliations:** 1 School of Medicine and Pharmacology, University of Western Australia, Fremantle Hospital, Fremantle, Western Australia, Australia; 2 Papua New Guinea Institute of Medical Research, Madang, Papua New Guinea; 3 Faculty of Health Sciences, Divine Word University, Madang, Madang Province, Papua New Guinea; 4 Infection and Immunity Division, Walter and Eliza Hall Institute, Parkville, Victoria, Australia; 5 Center de Recerca en Salut Internacional de Barcelona (CRESIB), Barcelona, Spain; Ege University, Turkey

## Abstract

**Background:**

There are few detailed etiologic studies of severe anemia in children from malaria-endemic areas and none in those countries with holoendemic transmission of multiple *Plasmodium* species.

**Methodology/Principal Findings:**

We examined associates of severe anemia in 143 well-characterized Papua New Guinean (PNG) children aged 0.5–10 years with hemoglobin concentration <50 g/L (median [inter-quartile range] 39 [33]–[44] g/L) and 120 matched healthy children (113 [107–119] g/L) in a case-control cross-sectional study. A range of socio-demographic, behavioural, anthropometric, clinical and laboratory (including genetic) variables were incorporated in multivariate models with severe anemia as dependent variable. Consistent with a likely trophic effect of chloroquine or amodiaquine on parvovirus B19 (B19V) replication, B19V PCR/IgM positivity had the highest odds ratio (95% confidence interval) of 75.8 (15.4–526), followed by *P. falciparum* infection (19.4 (6.7–62.6)), vitamin A deficiency (13.5 (5.4–37.7)), body mass index-for-age z-score <2.0 (8.4 (2.7–27.0)) and incomplete vaccination (2.94 (1.3–7.2)). *P. vivax* infection was inversely associated (0.12 (0.02–0.47), reflecting early acquisition of immunity and/or a lack of reticulocytes for parasite invasion. After imputation of missing data, iron deficiency was a weak positive predictor (6.4% of population attributable risk).

**Conclusions/Significance:**

These data show that severe anemia is multifactorial in PNG children, strongly associated with under-nutrition and certain common infections, and potentially preventable through vitamin A supplementation and improved nutrition, completion of vaccination schedules, and intermittent preventive antimalarial treatment using non-chloroquine/amodiaquine-based regimens.

## Author Summary

Severe anemia is associated with increased morbidity and mortality, and it is relatively common in developing countries. To establish the causes of severe anemia in a part of the world in which infection (especially malaria), malnutrition and genetic predisposition are prevalent, we studied children from Papua New Guinea who were hospitalized with severe anemia and healthy non-anemic community-based controls. Factors associated with severe anemia were parvovirus B19 infection, falciparum malaria, vitamin A deficiency, low body mass index for age and incomplete vaccination. Vivax malaria was inversely associated reflecting early acquisition of immunity or perhaps lack of circulating red blood cells for parasite invasion. Iron deficiency was only a weak positive predictor. These data show that severe anemia is multifactorial in Papua New Guinean children, strongly associated with under-nutrition and certain infections, and potentially preventable through vitamin A supplementation and improved nutrition, completion of vaccination schedules, and prevention or active treatment of malaria.

## Introduction

Severe anemia is a common reason for pediatric hospitalization in developing countries [1]–[4]. *Plasmodium falciparum* is the major cause in malaria-endemic areas [2], [5], [6], but nutritional deficiencies [7], [8], non-malarial infections [9], [10], and genetic conditions such as glucose-6-phosphate dehydrogenase deficiency (G6PD) [10] and other red cell polymorphisms [11] are also common and may contribute. Although the World Health Organization (WHO) has acknowledged the multifactorial nature of anemia [7], only one study has systematically examined the relative impact of, and interactions between, etiologic factors in a malaria-endemic country [1]. In this African case-control study [1], bacteremia, hookworm infestation, human immunodeficiency virus (HIV) infection, G6PD genotype, and deficiencies in vitamins A and B_12_ were significantly associated with severe anemia. Iron deficiency, considered the most common cause of anemia worldwide [8], was inversely associated, perhaps through protection against infection. In contrast to other studies performed in Africa [8], *P. falciparum* was only associated with severe anemia in children in urban areas with seasonal transmission but not in surrounding holoendemic rural sites [1].

Infections other than *P. falciparum* have varying effects on the prevalence of severe anemia. There have been inconsistent reports of associations between parvovirus B19 (B19V) and severe anemia [9], [12], [13] that might reflect differences in the intensity of use of chloroquine, a drug which has a trophic effect on B19V replication in bone marrow [14]. In tropical countries outside Africa, severe *P. vivax* infections most commonly present as severe anemia in West Papua [15] and yet *P. vivax* parasitemia appears to attenuate the post-treatment nadir in hemoglobin in Thai adults with co-existent *P. falciparum* infections [16].

Since available data suggest that specific epidemiologic settings will have their own hierarchy of causes underlying severe anemia, we carried out an etiologic study in Papua New Guinean (PNG) children with a high incidence of red cell polymorphisms such as alpha-thalassemia, exposure to infections with *P. falciparum*, *P. vivax* and B19V, and a high risk of micronutrient deficiencies.

## Methods

### Study site and local epidemiology

The study was performed in Madang Province on the north coast of mainland PNG. Most of the population of 450,000 are subsistence farmers and their families who live on lowland coastal plains. There is hyperendemic transmission of *P. falciparum* and *P. vivax* with approximately 50 infective bites per child per year [17], [18]. The countrywide HIV seroprevalence is 0.9% [19].

### Ethics approval

Approval for the study was obtained from the PNG Institute of Medical Research Institutional Review Board and the Medical Research Advisory Committee of the PNG Health Department. Written informed consent for participation was obtained from parent(s)/guardian(s) and, where possible, children gave assent to study procedures.

### Patients and controls

Children with severe anemia (hemoglobin concentration <50 g/L) were identified as part of an observational study of all children aged 0.5–10 years admitted to the pediatric ward of Modilon Hospital, the tertiary referral hospital for Madang Province, between October 2006 and November 2009 [20], [21]. Healthy non-anemic children (hemoglobin concentration >100 g/L), matched where possible by age and sex, were recruited as controls from community-based immunization clinics. They were asymptomatic and did not have i) a history of malaria within the previous fortnight, ii) current fever (axillary temperature >37.5°C), iii) respiratory distress (respiratory rate >40/minute plus in-drawing of chest wall or dyspnea), or iv) impaired consciousness (Blantyre Coma Score ≤4). The hemoglobin cut-points for severe anemia and non-anemic controls were selected on the basis of WHO-endorsed thresholds and those adopted in similar studies in other epidemiologic contexts [1], [2], [12], [22].

### Clinical procedures

A standardized case report form was completed by trained clinical research nurses who detailed each child's demographic details, history of current and/or past illness, examination findings, results of laboratory investigations, treatment and outcome. Vaccination history was identified from the health record book when this was available. It was assumed children without a documented vaccination history were unvaccinated. The expanded programme of immunization in PNG recommends two doses of vitamin A at 6 and 12 months [23]. Anthropometric z-scores for weight-for-age, height-for-age and body mass index (BMI)-for-age were calculated using WHO software [24], with a BMI-for-age z-score (BAZ) <2.0 considered indicative of wasting [24].

In the children with severe anemia, between 5 and 10 mL of venous blood were drawn if the clinical situation allowed, and 4–6 mL blood were collected from the healthy control children. Initial hemoglobin concentrations for cases and controls were measured using HemoCue (Angelholm, Sweden). Two skilled microscopists independently examined thick blood smears. Parasite density was calculated per 200 leukocytes using an assumed peripheral blood leukocyte count of 8000/µL. A senior microscopist adjudicated discrepant findings. Additional on-site tests in children with severe anemia comprised i) whole blood glucose (HemoCue, Ängelholm, Sweden) and lactate (Lactate Pro, Arkray, Japan) assay, ii) a full blood count (Coulter Ac·T diff, Beckman Coulter, Brea, USA), and iii) blood culture (BACTEC PEDS PLUS/F, Becton, Dickinson, Sparks, USA). Light-protected aliquots of plasma from all children were stored at −70°C prior to routine biochemical testing, B19V IgM and DNA assay, and measurement of serum vitamin A concentrations. Cell pellets were stored at −20°C for red cell folate assay, and host and parasite DNA extraction. Serologic testing for HIV, assays for G6PD enzyme activity and genotyping, and stool microscopy for intestinal parasites, were not performed.

Children found to have severe anemia were treated in accordance with PNG treatment guidelines [23], [25]. In addition to the routine use of iron/folate supplementation, and both antimalarial and antihelminthic treatment, transfusion of HIV-negative blood was available and recommended for all children with hemoglobin <40 g/L and for children with hemoglobin 40–50 g/L and signs of hemodynamic compromise [23], [25].

### Laboratory analyses

Plasma was assayed for concentrations of electrolytes, urea and creatinine, albumin and total protein, alanine aminotransferase (ALT), alkaline phosphatase (ALP), gamma glutamyl transferase (GGT), total bilirubin, calcium, phosphate, C-reactive protein (CRP), total cholesterol, triglycerides, creatine kinase, ferritin, soluble transferrin receptor (sTFR), vitamin B_12_. Other than vitamin B_12_ (Elecsys 2010, Roche Diagnostics, Mannheim, Germany) all biochemistry assays were performed on the COBAS INTEGRA 800 platform (Roche Diagnostics, Mannheim, Germany) using reagents supplied by the manufacturer. Red cell folate concentrations were measured using a 20 µL red cell pellet (Immulite 2000, Siemens Healthcare Diagnostic Ltd, Llanberis, United Kingdom). Vitamin A concentrations were measured by high performance liquid chromatography with UV detection (wavelength 325 nm) following protein precipitation and liquid-liquid extraction. An internal standard (retinol acetate) was used to correct for extraction recovery. All biochemical assays were monitored for accuracy and imprecision using appropriate internal quality control procedures as under the Quality Assurance Programme of the Royal College of Pathologists of Australasia and satisfying the requirements of external standards (ISO15089:2003). Vitamin B_12_, folate, vitamin A deficiencies were considered present if concentrations were <150 pmol/L, <260 pmol/L and <0.7 µmol/L, respectively. Iron deficiency was defined as a ratio of sTFR to the log_10_ of ferritin >5.6 [1].

### Parvovirus assays

Plasma was assayed for B19V IgM by EIA kit (Biotrin International) and for viral DNA using two specific oligonucleotide primers [9]. The presence of either detectable parvovirus B19-specific IgM or DNA was considered indicative evidence of recent infection [14].

### Genetic testing

DNA was extracted from 200 µL venous whole blood collected into EDTA anti-coagulant using QIAamp 96 DNA Blood Mini Kit (QIAGEN, Valencia, CA) and eluted in a final volume of 200 µL dH_2_O according to the supplier's instructions. We performed genotypic tests for red cell polymorphisms that are common in coastal Melanesian populations. These included 3.7-Kb and 4.2-Kb α-globin deletions associated with α+-thalassemia, a 27 bp deletion associated with South Asian Ovalocytosis (*SAO*), a 3 bp deletion in glycophorin C (*GLYC*) and genotyping of complement receptor-1 (*CR1*). The methods for the genotypic assays are detailed elsewhere [26]–[29].

### Data analysis

We used the statistical package R for all analyses [30]. Data were considered normally distributed if they passed the D'Agostino-Pearson test for normality. Bivariate comparisons between cases and controls were performed using the Student *t*-test or Mann-Whitney test for parametric and non-parametric continuous variables, respectively, or the Chi-squared test for dichotomous or nominal data. Associates of severe anemia were assessed using backward stepwise logistic regression analysis. Variables other than age were included based on biologic plausibility and *P*<0.10 on bivariate regression analysis and the most parsimonious model chosen based on Aikake's Information Criterion (AIC).

In conventional logistic regression, a missing value for a single variable means that other valid data for an individual are lost. *Ad hoc* methods such as replacing a missing value with a sample mean or median creates potential for bias. An alternative approach is multiple imputation (MI) in which each missing value is imputed a number of times (commonly five) using informative prior knowledge of the distribution (categorical, nominal or continuous) of each variable. MI generates a number of complete datasets that facilitate refinement of logistic regression models through comparisons of AIC and statistical testing [31]. Although data were available from >90% of participants for most variables, some datasets were incomplete due to factors such as difficulty with venesection or assay failure. We imputed these data using the program AMELIA [32]. Briefly, each variable was defined as categorical, nominal or continuous. Non-parametric, continuous variables were log-transformed and intuitive constraints placed on the possible output data. For example, values for measured analytes could not be <0. Following imputation, AMELIA provides visual and statistical diagnostics that ensures that the imputed data are representative of measured data. Missing data for cases and controls were imputed separately. Logistic regression modelling was performed on each of the five completed imputed datasets and the final adjusted odds ratios (ORs) determined by calculating the mean from each model [31]. After combination of the five imputed datasets, the partial attributable risks for multiple exposure factors and confounders were estimated using the R package pARccs with confidence intervals estimated using a non-parametric bootstrapping procedure [33], [34].

## Results

### Patients and controls

**Figure 1:**
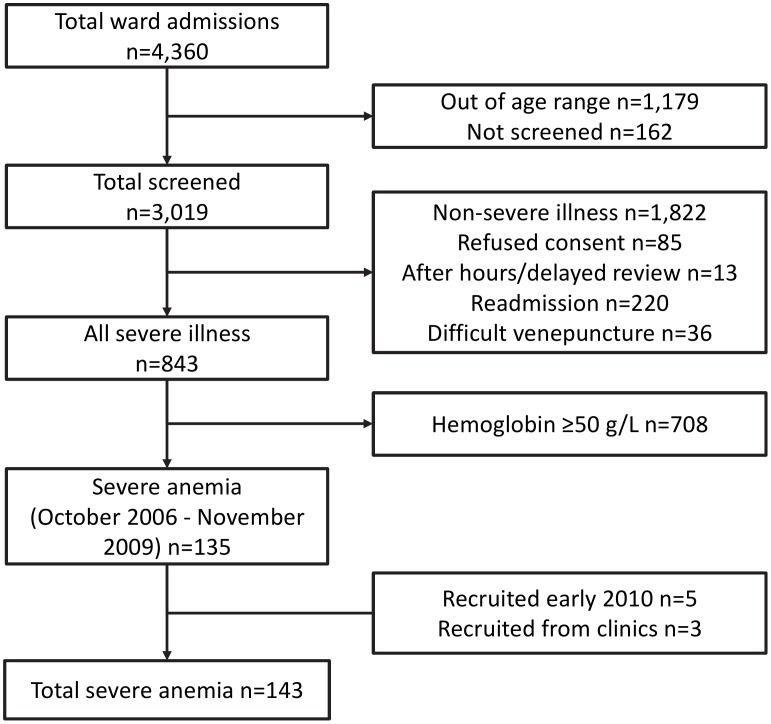
Consort diagram describing the recruitment process of children with severe anemia.

One hundred and forty three children with severe anemia were recruited (see Figure 1). In the period from October 2006 to the end of 2009, 135 of 3019 (4.5%) children admitted to Modilon Hospital had severe anemia. Five further severely anemic children were recruited between January and May 2010 using passive surveillance after the larger study of severe illness had been completed, and three additional severely anemic children were opportunistically identified at immunization clinics where the 120 non-anemic healthy controls were enrolled. The median [inter-quartile range] (IQR) hemoglobin in the severe anemia group was 39 [33–44] g/L compared with 113 [107–119] g/L in the control group.

The demographic and anthropometric features of cases and controls are summarized in Table 1. There were no significant differences in age, sex distribution, ethnicity or adoption between cases and controls. The controls were significantly more likely to use bednets and to have completed vaccinations, consistent with better maternal education. Although height was similar in the two groups of children, those with severe anemia had significantly lower body weights, mid upper arm circumference and BAZ than the non-anemic controls.

**Table 1:** Demographic and anthropometric characteristics of severely anemic children and healthy non-anemic controls.

	Cases	Controls	*P*-value
Number	143	120	
Sex (males)	50.3	47.5	0.65
Age (months)	38 [24–61]	42 [29–57]	0.26
Maternal education			
None/elementary	36.7	20.9	
Primary	53.9	63.5	0.027
Secondary or tertiary	9.4	15.7	
Ethnicity (Madang or Sepik)	84.6	90.0	0.27
Adopted	8.4	5.8	0.48
Bednet use	82.6	94.0	0.007
Incomplete vaccination	49.0	30.0	0.001
Height (cm)	87 [77–100]	90 [80–101]	0.19
Weight (kg)	11 [9.0–14.5]	12.5 [11.0–15.7]	<0.001
Mid upper arm circumference (cm)	14 [13–15]	15 [14–15.5]	<0.001
Body mass index to age Z-score (BAZ)	−0.58 [−1.99–0.55]	0.01 [−1.12–1.22]	0.009
Wasting (BAZ <2)	25.9	12.3	0.01

Data are percentages or median and [inter-quartile range].

The laboratory features of cases and controls are summarized in Table 2. The severely anemic children were more likely to have falciparum malaria and with a parasite density greater than that of parasitemic controls. Serum vitamin B_12_, folate and vitamin A concentrations were all significantly lower in the severe anemia group and more of these children were deficient in each case. Serum vitamin A concentrations were higher in those children who had received both doses of vitamin A when compared to one or no doses (0.68 µmol/L [IQR 0.42–0.89] vs 0.57 [0.37–0.79], *P* = 0.06). Serum ferritin concentrations were, consistent with serum CRP concentrations and the clinical features, higher in the severe anemia group.

**Table 2:** Biochemical, hematologic and genetic characteristics of severely anemic children and non-anemic controls.

	Number (%) with available data	Cases	Controls	*P*-value
		n = 143	n = 120	
*Plasmodium falciparum* parasitemia	263 (100)	58.7	5.8	<0.001
>1,000/µL (% of *P. falciparum* positives)		77.8	0.8	<0.001
*Plasmodium vivax* parasitemia	263 (100)	4.2	19.2	0.001
>500/µL (% of *P. vivax* positives)		33.3	43.5	1.00
Serum vitamin B_12_ (pmol/L)	242 (92)	298 [207–406]	375 [301–455]	<0.001
Vitamin B_12_ deficiency (<150 pmol/L)		9.0	0.8	0.005
Serum folate (pmol/L)	212 (81)	542 [369–716]	634 [469–863]	0.01
Folate deficiency (<260 pmol/L)		9.5	1.7	0.01
Serum vitamin A (µmol/L)	244 (93)	0.40 [0.28–0.58]	0.82 [0.66–0.94]	<0.001
Vitamin A deficiency (<0.7 µmol/L)		73.7	21.5	<0.001
Serum ferritin (mg/L)	243 (92)	369 [239–802]	49 [34–71]	<0.001
Low ferritin (<10 mg/L)		4.8	0.8	0.12
Soluble transferrin receptor (mg/L)	243 (92)	7.5 [5.0–12.6]	6.1 [5.0–7.1]	0.001
Soluble transferrin receptor/log ferritin	243 (92)	2.9 [1.8–5.0]	3.6 [2.9–4.3]	0.009
Iron deficiency (sTFR/log ferritin >5.6)		18.7	10.9	0.089
Serum bicarbonate (mmol/L)	248 (94)	16.3 [15.0–18.2]	16.4 [15.0–17.4]	0.606
Blood lactate (mmol/L)	132 (92)	3.1 [2.1–4.6]	-	-
Serum creatinine (µmol/L)	252 (96)	24 [19–31]	24 [20–26]	0.657
Serum bilirubin (µmol/L)	253 (96)	11.2 [6.5–21.6]	3.4 [2.7–4.1]	<0.0001
Serum alanine aminotransferase (IU/L)	250 (95)	13 [10–19]	12 [9–17]	0.053
Serum C-reactive protein (mg/L)	248 (94)	42 [14–114]	1.4 [3.6–2.7]	<0.0001
Parvovirus B19 PCR positive	263 (100)	15.4	1.7	<0.0001
Parvovirus B19 positive IgM	263 (100)	14.8	1.7	<0.0001
Parvovirus B19 PCR or IgM positive	263 (100)	23.8	2.5	<0.0001
South Asian Ovalocytosis (Δ27bp deletion)	239 (91)	7.4	3.4	0.256
Glycophorin C Δex3bp deletion	223 (85)			
Deletion/deletion		12.7	5.4	
Deletion/wildtype		36.3	38.7	0.220
Wildtype/wildtype		60.8	55.9	
Complement receptor 1 polymorphism	230 (87)			
AA		13.4	8.1	
AG		36.1	35.1	0.368
GG		50.4	56.8	
Alpha-thalassemia (3.7 or 4.2 kb deletions)	171 (65)			
Wildtype/wildtype		24.7	22.9	
Deletion/wildtype		25.8	40.5	0.104
Deletion/deletion		49.4	35.1	

Data are percentages or median and [inter-quartile range].

Due to difficulty with venesection, occasional parenteral concerns about the amount of blood to be taken from an unwell child, and prioritisation of on site and other tests requiring relatively small volumes, there was sufficient blood for culture in only 100 children in the severe anemia group (70%). In one of these children, an isolate identified as a *Klebsiella spp.* was cultured after 5 days of hospitalization and was associated with disseminated ascariasis. A second child with severe anemia had acute bacterial meningitis with *Hemophilus influenzae* type b isolated from CSF. No other invasive bacterial infections were identified.

### Independent risk factors for severe anemia

The summary of the most parsimonious logistic regression model is shown in Table 3. The presence of B19V had the highest OR of 75.8, and *P. falciparum*, vitamin A deficiency, wasting and incomplete vaccination were also independently associated with severe anemia. The presence of *P. vivax* was negatively associated with severe anemia. Although included in the initial backward stepwise logistic regression model, maternal education, mosquito bednet use, red cell folate deficiency, vitamin B_12_ deficiency and alpha-thalassemia genotype did not prove to be independent associates of severe anemia. Iron deficiency was also not significantly associated with severe anemia. When this variable was forced into the most parsimonious model, there was a trend to significance (OR 2.7 [0.9–8.5], *P* = 0.07).

**Table 3:** Summary of logistic regression model for severe anemia.

	Odds ratio (95% CI)	*P*-value
Parvovirus B19 infection	75.8 (15.4–526)	<0.0001
*Plasmodium falciparum* infection	19.4 (6.7–62.6)	<0.0001
*Plasmodium vivax* infection	0.12 (0.02–0.47)	0.0055
Vitamin A deficiency	13.5 (5.4–37.7)	<0.0001
Wasting (BAZ <2)	8.4 (2.7–27.0)	0.0003
Incomplete vaccination	2.94 (1.3–7.2)	0.0151

Severe anemia was multifactorial in the majority of children. Only three of 112 severely anemic children with complete datasets did not have at least one independent risk factor compared with 34 of 113 in the control group, whilst 88.3% and 15.1% of cases had at least two and four risk factors for severe anemia, respectively. After performing MI, logistic regression modelling was performed on each of the five complete datasets. The ORs for each of the variables in each model and the mean values across all 5 models are presented in Table 4. The mean ORs and 95% CI across the five datasets were of similar magnitude to those of the logistic regression in Table 3 for B19V, *P. falciparum* infection, *P. vivax* infection, vitamin A deficiency, wasting, and incomplete vaccination. Additionally, all logistic regression models using imputed data included iron deficiency as an independent associate.

**Table 4:** Odds ratios from logistic regression of imputed datasets.

Imputed dataset	1	2	3	4	5	MI Average	Partial attributable risk (% (95% CI))
Parvovirus B19	60.7	63.3	60.1	52.3	56.2	58.5 (53.1–63.9)	10.4 (8.2–12.8)
*Plasmodium falciparum*	19.1	16.1	18.0	17.7	19.7	18.1 (16.4–19.9)	24.5 (22.1–26.8)
*Plasmodium vivax*	0.13	0.11	0.11	0.11	0.10	0.11 (0.10–0.13)	
Vitamin A deficiency	10.8	12.8	10.7	10.7	13.1	11.6 (10.1–13.1)	36.0 (31.2–39.2)
Wasting (BAZ <2)	5.55	6.99	6.74	5.41	5.81	6.1 (5.2–7.0)	8.9 (6.5–11.2)
Incomplete vaccination	2.20	2.22	1.89*	1.99*	2.2*	2.1 (1.9–2.3)	9.8 (4.6–13.1)
Iron deficiency	3.68	2.91	3.92	3.92	4.13	3.7 (3.1–4.3)	6.4 (4.7–8.0)

Multiple imputation (MI) averages and 95% confidence intervals (CI) are given, together with partial attributable risks and boot strap 95% CI.

The significant independent risk factors accounted for 96.0% of population attributable risk. The partial attributable risks for B19V, *P. falciparum*, vitamin A deficiency, wasting and incomplete vaccination were calculated using the R package ‘pARccs’ with *P. vivax* included as a confounder. In this analysis, nutritional deficits accounted for 51.3% of attributable risk, specifically vitamin A deficiency (36.0%), wasting (8.9%) and iron deficiency (6.4%). *P. falciparum* and B19V accounted for 24.5% and 10.4%, respectively. Incomplete vaccination had a partial attributable risk of 9.8%.

### Features of severe anemia in falciparum vs non-falciparum malaria

The demographic, clinical, laboratory and genetic features of the 84 severely anemic children with falciparum malaria (58.7%) and those without are shown in Table 5. Vitamin A deficiency was more common in the children with falciparum malaria, a group in which almost all were deficient (96.0% vs 59.2%, *P*<0.0001). Iron deficiency (8.2% vs 42.5%, *P* = 0.0006) and B19V (15.5% vs 33.9%, *P* = 0.014) were less common. The median red cell distribution width (RDW) was lower in children with falciparum malaria (median [IQR] 19 [17–24] vs 26 [21–30], *P*<0.0001). Serum bilirubin and CRP concentrations were also higher in this group. Of the red cell polymorphisms tested, those with a homozygote genotype for the *GLYCΔex3bp* deletion were under-represented amongst the severely anemic children with falciparum malaria (5.6% vs 22.5%, *P* = 0.02).

**Table 5:** Clinical, laboratory and genetic features of children with severe anemia by *Plasmodium falciparum* infection status.

	*P. falciparum*	Non-*P. falciparum*	*P*-value
	(n = 84)	(n = 59)	
Sex (males)	45.2	57.6	0.18
Age (months)	36 [25–52]	41 [24–80]	0.11
Maternal education			
None/elementary	30.8	42.6	
Primary	57.7	44.4	0.25
Secondary or tertiary	9.0	13.0	
Ethnicity (Madang or Sepik)	84.5	84.7	0.93
Adopted	11.4	5.6	0.30
Bednet use	83.8	81.0	0.82
Incomplete vaccination	51.2	50.8	1.00
Wasting (BAZ <2)	21.3	32.1	0.17
Vitamin B12 deficiency (<150 pmol/L)	11.0	6.1	0.52
Folate deficiency (<260 pmol/L)	13.0	4.9	0.29
Vitamin A deficiency (<0.7 µmol/L)	96.0	59.2	<0.0001
Iron deficiency (sTFR/log ferritin >5.6)	8.2	42.5	0.0006
Parvovirus B19 PCR/IgM positive	15.5	33.9	0.014
Red cell distribution width	19 [17–24]	26 [21–30]	<0.0001
Serum bicarbonate (mmol/L)	16.2 [15.0–17.9]	16.5 [15–18.5]	0.48
Blood lactate (mmol/L)	3.3 [2.2–5.1]	3.1 [2.1–4.2]	0.14
Serum creatinine (µmol/L)	24 [19–31]	25 [18–32]	0.81
Serum bilirubin (µmol/L)	15.1 [8.6–24.9]	7.4 [4.4–12.7]	0.0002
Serum alanine aminotransferase (IU/L)	14 [11–19]	11 [8–19]	0.02
Serum C-reactive protein (mg/L)	93 [36–151]	15 [5–42]	<0.0001
Southeast Asian ovalocytosis (Δ27bp deletion)	5.3	11.1	0.29
Glycophorin C Δex3bp deletion			
Deletion/deletion	5.6	22.5	
Deletion/wildtype	33.3	32.5	0.02
Wildtype/wildtype	61.1	45.0	
Complement receptor 1 polymorphism			
AA	9.5	20.0	
AG	37.8	33.3	0.27
GG	52.7	47.7	
Alpha-thalassemia (3.7 or 4.2 kb deletion)			
Wildtype/wild type	21.9	30.3	
Deletion/wild type	25.0	27.3	0.56
Deletion/deletion	53.1	42.4	

Data are percentages or median and [inter-quartile range].

### Clinical course

Four (2.8%) children with severe anemia died. Two were comatose on admission and had cerebral malaria in addition to severe anemia. One had acute renal failure and metabolic acidosis due to *P. vivax.* The fourth child died following a prolonged illness accompanied by wasting, lymphadenopathy and hepatosplenomegaly. After failing empiric therapy for tuberculosis, a presumptive clinical diagnosis of lymphoma was made.

## Discussion

The present study shows that severe anemia is common and multifactorial in PNG children. Parvovirus B19 infection, falciparum malaria, vitamin A deficiency, wasting and incomplete vaccination were the main etiologic factors, while *P. vivax* infection was negatively associated with severe anemia. The presence of iron deficiency was predictive in our children only after imputation of missing data, with a relatively small overall contribution to total attributable risk. Although vitamin B_12_ and folate deficiency were present in 9.0% and 9.5%, respectively, of our severely anemic children, neither was independently associated with severe anemia, which was also the case for adoption, ethnicity, maternal education, bednet use and all of the red cell polymorphisms assessed. The causes of severe anemia in PNG children can, therefore, be considered as comprising two main categories, namely under-nutrition (vitamin A deficiency and wasting) and infection (falciparum malaria, B19V infection and perhaps incomplete vaccination), which are both associated with a mainly hypoproliferative anemia [35], [36].

Epidemiologic studies have shown an association between low serum vitamin A and anemia in different populations [37], [38]. While the hematologic phenotype of vitamin A deficiency-associated anemia is incompletely characterized, impaired erythropoiesis, reduced immunity to infection, and modulation of iron metabolism are potential underlying mechanisms [38]. Wasting may be a surrogate for deficiencies in other nutrients that are important for normal bone marrow function. Although there were no independent associations between iron, vitamin B_12_ and folate deficiency and severe anemia in the present study, it is possible that these might act in concert with deficiencies in vitamins B_6_ and E, riboflavin, zinc and copper to substantially reduce erythropoiesis in our under-nourished children [36].

In contrast to the African case-control study [1], we did not find that iron deficiency protected against severe anemia in our children. Indeed there was the suggestion that it was a minor positive contributor. The authors of the African study used a multivariate structural model to implicate reduced infection as a major consequence of iron deficiency that helped prevent severe anemia [1]. Although blood cultures were only available for 70% of our children with severe anemia and were not taken from any healthy controls, we found only two children with evidence of invasive bacterial infection. This is contrast to the 15% of cases and 4% of controls with confirmed infection in the Malawian study [1]. This difference in the risk of bacterial infection emphasises the need for caution in extrapolating the results of observational studies from a particular epidemiologic situation.

We found opposing associations between *P. falciparum* and *P. vivax* parasitemias and severe anemia. As in most studies conducted in sub-Saharan Africa [2], [5], [6], there was a strong positive association with concurrent *P. falciparum* infection in our PNG children. This reflects the increase in incidence of falciparum malaria during first three years of life in PNG children living in holoendemic areas [39] together with a significant subsequent risk of infection in later childhood [17]. By contrast, acquisition of immunity to *P. vivax* in this epidemiologic situation is rapid, such that children have almost complete clinical immunity by the age of 5 years [17]. Although asymptomatic *P. vivax* infections are common in PNG children aged 2–10 years, they are associated with only small decreases in hemoglobin [40] that are unlikely to contribute significantly to the risk of severe anemia in this age group. In neighbouring West Papua, Indonesia, *P. vivax* is an important cause of severe anemia in very young infants with an excess risk over that associated with *P. falciparum* [41]. However, while the present study limited enrolment children aged >6 months, only five of the children with severe anemia (3.5%) were aged <12 months (four with falciparum malaria and one with vivax malaria), implying that we were unlikely to have missed significant numbers of infants with severe anemia outside the first few months of life.

An alternative explanation for the inverse relationship between severe anemia and *P. vivax* reflects the fact that this parasite invades young red cells, especially reticulocytes, which are absent or present in low numbers in established severe hypoproliferative anemias [35], [36], [42], [43]. This means that vivax malaria cannot readily develop in severely anemic children irrespective of the cause, with or without a contribution of *P. vivax* itself to the hypoproliferative state [44]. Although severe anemia in our children may not support a *P. vivax* parasitemia detectable by light microscopy, it does not exclude a significant prior contribution. The importance of antecedent parasitemia as an independent determinant of anemia has been highlighted in an African study of falciparum malaria and acute and relapsing vivax malaria might be an even greater cause of a subsequent presentation with severe anemia [45].

Cross-sectional and longitudinal surveys in PNG and neighbouring Vanuatu have shown negative correlations between different *Plasmodium* species [46], [47], and mixed species infection tend to be substantially less common among symptomatic compared to asymptomatic infections [47]. Epidemiologic evidence from Thailand has also shown that the rate of severe malarial disease, but not mortality, is lower in patients with *P. falciparum* when there is a mixed infection with *P. vivax* [48]. In addition, co-infection with *P. vivax* appears to abrogate the nadir hemoglobin resulting from malaria due to *P. falciparum* [16]. This has been interpreted as indicating that *P. vivax* provides protection against *P. falciparum* clinical disease [48], [49]. The observed negative association between species could, however, equally be the result of *P. vivax* being suppressed in severe *P. falciparum* malaria, either directly by *P. falciparum* [50] or by the innate host response [51].

The children with severe anemia and falciparum malaria were more likely to be vitamin A deficient than the severely anemic aparasitemic children, but it is unclear whether this represents a cause or consequence of malaria infection [37]. Children with severe anemia due to *P. falciparum* also had a lower median RDW, a measure that has been associated with erythropoietic activity where reticulocyte data are unavailable [52]. This finding is consistent with an attenuated erythropoietic response in severe anemia due *P. falciparum* [53]. The only significant finding relating to red cell polymorphisms in the present study was an under-representation of the homozygote genotype for the *GLYCΔex3bp* deletion in the severely anemic *P. falciparum* group, but this association has not be observed consistently in other studies from PNG [28].

Our data confirm and extend the body of evidence implicating B19V as a cause of severe anemia in PNG. Although it had the highest OR (75.8) for an individual, it accounted for only 10% of the population attributable risk. In a previous study of severe anemia in PNG children, B19V and the effects of malaria were the only etiologic factors considered [9]. One explanation for the variable association between B19V and severe anemia in reports to date [9], [12], [13] is the trophic effects of chloroquine and amodiaquine on the replication of B19V in bone marrow [14]. Associations between severe anemia and B19V have been shown in countries such as PNG where these drugs have been deployed widely and not where alternatives such as sulfadoxine-pyrimethamine have been used [14]. It is possible that the association between B19V and severe anemia may disappear as artemisinin combination therapy (ACT) replaces chloroquine/amodiaquine-based regimens.

Our study had limitations. Due to socio-cultural barriers to testing, HIV serology was not performed. Despite being a risk factor for severe anemia in African children [1], PNG has a relatively low prevalence of HIV seropositivity (0.9%) that would be unlikely to contribute to severe anemia at a population level [19]. Stool examination for intestinal helminths was not performed for logistic reasons, but the contribution of heavy worm burden to severe anemia is likely to be via nutrient deficiencies and/or malnutrition, both of which we incorporated in our analyses. The choice of appropriate controls in such studies can be difficult. We aimed to identify the most important etiologic factors in severe anemia by comparing the severe anemia cases with children with an optimal hemoglobin concentration for this epidemiologic setting. The concept of ‘optimal’, ‘normality’ or ‘healthy’ in developing countries has been considered philosophical but can be defined as ‘not suffering significant illness’ or being in ‘reasonable health’ [54]. Our controls conformed to this definition. Conversely, the inclusion of children with non-optimal, intermediate hemoglobin concentrations of 50–100 g/L (as was done in the Malawian study [1]) could also be a source of bias, potentially obscuring clinically relevant contributors to severe anemia. Nevertheless, by utilizing a non-anemic, hemoglobin threshold of >100 g/L, the independent associations identified may have been different to those reported in other studies in which healthy community controls have had median hemoglobin concentrations <100 g/L [1], [2], [9].

The present study confirms that severe anemia in PNG children is multi-factorial and suggests that vitamin A deficiency and *P. falciparum* infection are the most important contributors. Vitamin A supplementation has been shown to reduce malaria infection [55], [56] but may have other beneficial effects on hematopoiesis [38] that contribute to reduced all-cause mortality [57], [58]. Currently, vitamin A is given as part of the expanded program of immunization in PNG at 6 and 12 months of age. Incomplete vaccination was an independent predictor of severe anemia in our children and so current efforts to up-scale supplementary immunization every 2–3 years should have benefits for the incidence of anemia, especially if vitamin A supplementation were included. Other strategies to improve childhood nutrition would also be beneficial in PNG children. Implementation of intermittent preventive treatment in infancy could reduce the burden of anemia [59], especially if ACT replaces chloroquine/amodiaquine-based regimens [14].

## Supporting Information

Checklist S1 **STROBE checklist of items that should be included in reports of cross-sectional case-control observational studies with detailed referencing of requirements to the text of the paper.**
(DOC)
